# New citizen science initiative enhances flowering onset predictions for fruit trees in Great Britain

**DOI:** 10.1093/hr/uhae122

**Published:** 2024-04-23

**Authors:** Chris Wyver, Simon G Potts, Richard Pitts, Mike Riley, Gerard Janetzko, Deepa Senapathi

**Affiliations:** Centre for Agri-Environmental Research, School of Agriculture, Policy and Development, University of Reading, Reading RG6 6AR, UK; Centre for Agri-Environmental Research, School of Agriculture, Policy and Development, University of Reading, Reading RG6 6AR, UK; Oracle for Research, Oracle Corporation UK Ltd., Oracle Parkway, Thames Valley Park (TVP), Reading RG6 1RA, UK; Oracle for Research, Oracle Corporation UK Ltd., Oracle Parkway, Thames Valley Park (TVP), Reading RG6 1RA, UK; Oracle for Research, Oracle Corporation UK Ltd., Oracle Parkway, Thames Valley Park (TVP), Reading RG6 1RA, UK; Centre for Agri-Environmental Research, School of Agriculture, Policy and Development, University of Reading, Reading RG6 6AR, UK

## Abstract

Accurately predicting flowering phenology in fruit tree orchards is crucial for timely pest and pathogen treatments and for the introduction of managed pollinators. Making predictions requires large datasets of flowering dates, which are often limited to single locations. Consequently, the resulting phenology predictions are not representative across larger geographic areas. Citizen science may offer a solution to this data gap, with millions of biological records across a wide range of taxa recorded annually. Here, a new citizen science platform called ‘FruitWatch’ is introduced, monitoring the flowering dates of fruit trees in Great Britain. The objectives of this study are to assess the suitability of FruitWatch submissions to (i) detect latitudinal variation in flowering onset dates, (ii) parameterize existing phenology modelling frameworks, and (iii) make predictions of flowering onset dates across Great Britain for a single year. Using data for four cultivars from 2022, linear models reveal significant latitudinal delays in flowering onset of as much as 1.49 ± 0.63 days per degree latitude further north (Pear ‘Conference’), with significant delays also seen in Cherry ‘Stella’ (1.39 ± 0.48 days) and Plum ‘Victoria’ (1.22 ± 0.18 days). FruitWatch informed phenology modelling frameworks performed well for predicting flowering onset, with root mean square error values of predictions from validation datasets ranging between 4.6 (‘Victoria’) and 8.0 (‘Conference’) days. The parameterized models also provided realistic flowering onset predictions across Great Britain in 2022, with earlier flowering dates predicted in warmer areas. These findings demonstrate the potential of citizen science data to offer growers cultivar- and location-specific phenology predictions to help inform orchard management.

## Introduction

It is well documented that climate change is having a diverse range of impacts on many taxa. One of these impacts is changes in phenology, defined here as the ‘timing of recurrent biological events’ [1], and there is mounting evidence to suggest that many species have exhibited phenological shifts in response to the changing climate. These shifts have been reported, across a range of taxa, including plants [2], insects [3], and birds [4].

Plants, particularly fruit trees are of both commercial and public interest in Great Britain, including pome fruits such as apples (*Malus domestica*) and pears (*Pyrus communis*), and stone fruits such as cherries (*Prunus avium*) and plums (*Prunus domestica*). Pome and stone fruits are an economically important horticultural crop in Great Britain, with 239 300 tonnes of apples, 17 900 tonnes of pears, 6300 tonnes of plums, and 4100 tonnes of cherries (plus an additional 170 100 tonnes of cider apples and perry pears) grown in the UK (encompassing Great Britain and Northern Ireland) in 2022 [5]. This production has an estimated total value of over £600 million [5], making pome and stone fruits very important sectors in Great Britain’s agricultural industry, and so any climate driven shifts in fruit tree biology are important to understand.

Identifying phenological shifts across flora and fauna usually requires long-term datasets, collected either through systematic or opportunistic observations, or through analysis of museum collections, and the current knowledge of phenological shifts of pome and stone fruit crops in Great Britain is limited. Long-term data that does exist comes primarily from single focal orchards. This includes the National Fruit Collection, located in Brogdale, Kent, UK, which holds phenological records dating back to the 1960s. Data from this collection show advances in flowering dates of both apples, likely linked to changing climatic conditions [6] and pears [7]. From further afield, cherries and plums have also been shown to be advancing flowering phenology. Research carried out in German sweet cherry orchards highlights advances in flowering onset dates of 2.0 days per decade [8], and Norwegian plums exhibit a 3.2 day per decade advance in full bloom dates [9].

While these long-term, single-location, or few-location datasets provide valuable insights into phenological shifts within these specific locales, they come with the trade-off that they may not be generalisable over space. Phenology may be influenced by specific local environmental and management conditions unique to a location, including cultivar selection [7], soil composition [10] and microclimate [11]. These different influences may be disentangled by incorporating citizen science to increase the spatial scale of the dataset, and therefore increase its geographical applicability.

Citizen science, defined as ‘the involvement of non-professionals in scientific investigations’ [12], can be either systematic (with a standardized survey procedure) or opportunistic (ad hoc recordings) and has often been used in place of data collected by traditional scientific protocols to detect phenological change in a wide range of taxa, including forest trees [13], bumblebees [14], plants [15], and birds [16].

**Table 1:** Number of records of each cultivar used in the analysis

Cultivar	Total records	Unique grid squares	Model calibration records	Model validation records
Apple ‘Bramley’	64	54	41	13
Cherry ‘Stella’	25	20	15	5
Pear ‘Conference’	118	92	69	23
Plum ‘Victoria’	242	178	133	45

Citizen science and biological recording are popular activities for many people, especially in Great Britain. This includes both systematic schemes, such as the Butterfly Monitoring Scheme (www.ukbms.org), and ad hoc schemes such as Nature’s Calendar (www.naturescalendar.woodlandtrust.org.uk). As a result, large citizen science datasets—both systematically and opportunistically collected—exist for a wide range of taxa, including bees, hoverflies, birds, and many plant and tree species. A notable exception to this list is pome and stone fruits, including apples, pears, cherries, and plums. All four of these trees are grown extensively across Great Britain, both for commercial and noncommercial purposes, and can be found in many public (e.g. community orchards, stately homes) and private (e.g. commercial orchards, private gardens) places.

Given the prevalence of these fruit trees in the British landscape, there arises a potential opportunity to recruit citizen scientists to record flowering dates, and to begin to understand phenological patterns beyond the focal orchards with long-term data for many cultivars. Additionally, as pome and stone fruits are grown across Great Britain, in a wide variety of climate conditions, they provide a good system for a space-for-time substitution study, allowing for testing of the sensitivity of flowering dates to different climate conditions in the absence of a long-term dataset.

Understanding the phenology of pome and stone fruit trees, particularly during the flowering stage is key to assessing phenological synchrony, and identifying any mismatches that are arising with pollinators and pests. Maintaining synchrony with pollinators is of particular importance for apples, pears, cherries, and plums, as they depend on insect pollination to set fruit [17]. Many cultivars of all four crops require cross-pollination from a suitable polliniser cultivar to produce more and/or higher quality fruit [18–20]. Much of this cross-pollination is carried out by insects, including managed honeybees, bumblebees, solitary bees, and hoverflies being often cited as major contributors [17, 18]. Therefore, maintaining temporal synchrony, both with the polliniser cultivars and the insect pollinators is crucial to maximize fruit set, quality, and ultimately economic value. Recent reports have shown phenological shifts in pollinators, such as wild bees [3, 6] and hoverflies [21], and therefore understanding phenological trends in fruit tree flowering is critical for understanding potential disruptions in temporal synchrony between flowering and insect pollinators.

This study introduces a novel citizen science platform, called FruitWatch (www.fruitwatch.org)—a collaborative effort between researchers at the University of Reading and Oracle for Research, which was launched before the British fruit tree flowering season in 2022, and asks citizen scientists to record flowering dates of apple, pear, cherry, and plum trees. The data generated through the FruitWatch platform tracks phenological shifts in the four focal crops, initially using space-for-time substitution to assess the effect of climate on flowering dates and using a case study of the most recorded cultivar of each tree type to assess whether flowering dates of particular cultivars show different sensitivities to climate. The specific research questions addressed here are:

1. Does flowering onset phenology shift across a latitudinal gradient in Great Britain, across a single year?
2. Can citizen science data be used to parameterize existing phenology models in a space-for-time substitution?
3. How well can models parameterized using a single year of citizen science data predict flowering onset dates across fruit-growing areas in that year?

## Results

### Does flowering onset phenology vary across a latitudinal gradient in Great Britain, across a single year?

**Figure 1:**
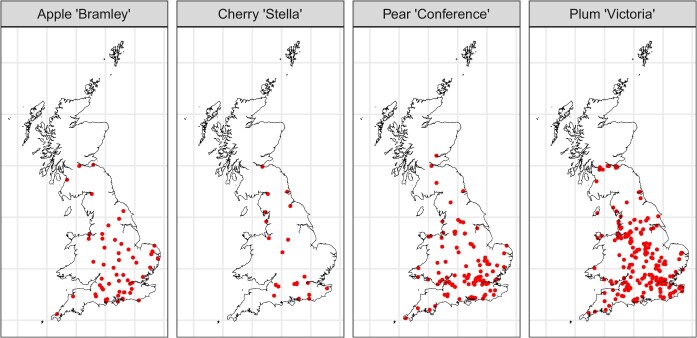
Geographic location of flowering onset record submissions for each cultivar

Across the four cultivars, a total of 449 verified and validated records were received in 2022. These records had a good geographical spread across populated areas of Great Britain. The split of these records can be found in Table 1 and the locations in Fig. 1.

**Figure 2:**
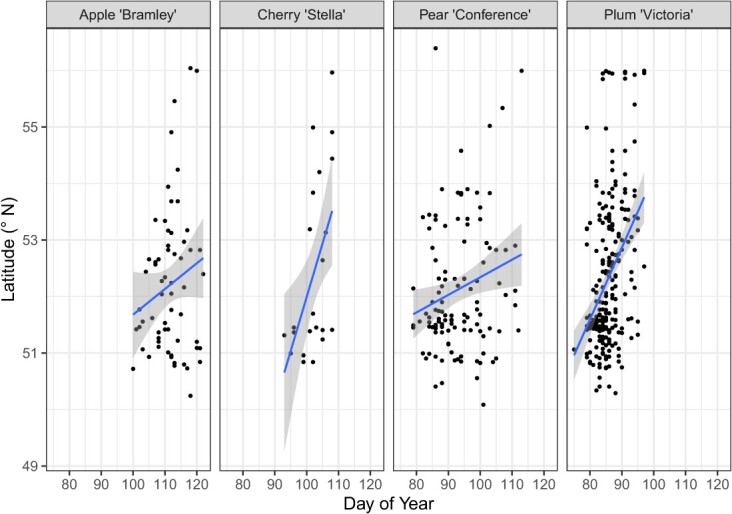
Relationship between latitude and flowering onset date for 2022. Shaded area represents 95% confidence interval

Linear mixed-effects models to assess geographical trends in phenology revealed significantly delayed flowering onset phenology at more northerly latitudes for ‘Stella’, ‘Conference’, and ‘Victoria’, ranging from 1.22 ± 0.18 days per ° of latitude further north (‘Victoria’) to 1.49 ± 0.63 days (‘Conference’) (Table 2, Fig. 2).

**Table 2:** Linear model output assessing the variation in flowering onset date across latitudes

Cultivar	Estimate	SE	Statistic	Corrected *p*-value
Apple ‘Bramley’	0.79	0.52	1.51	0.135
Cherry ‘Stella’	1.39	0.48	2.88	0.017
Pear ‘Conference’	1.49	0.63	2.37	0.025
Plum ‘Victoria’	1.22	0.18	6.65	< 0.001

Positive estimates indicate delayed phenology at higher latitudes.

### Can citizen science data be used to parameterize existing phenology models in a space-for-time substitution?

**Figure 3:**
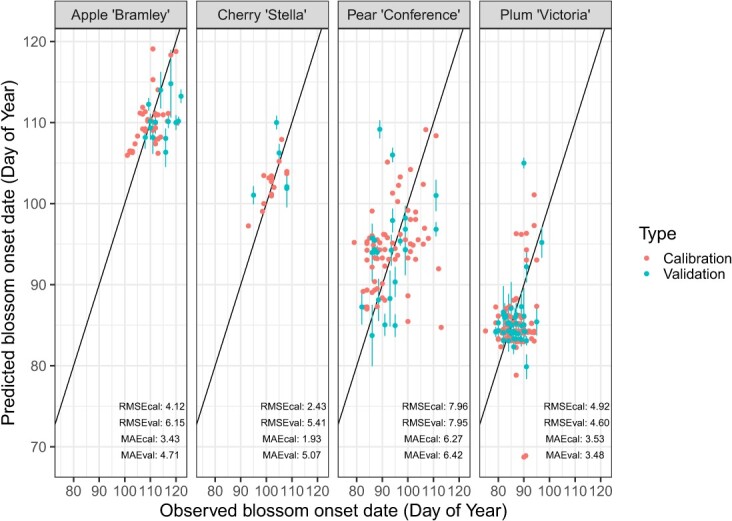
Observed versus predicted flowering onset dates for calibration and validation data sets and model performance metrics for each cultivar. Black line indicates perfect agreement between observed and predicted flowering dates. Error bars represent the 16th and 84th percentiles of standard deviation across the 10 bootstrapping iterations within the validation dataset.

The PhenoFlex modelling framework produced RMSE values for the calibration dataset of between 2.4 (Stella) and 8.0 days (Conference), and MAE values of between 1.9 days (Stella) and 6.3 days (Conference). RMSE values for the validation dataset also varied, ranging from between 4.6 days (Victoria) and 8.0 days (Conference), and MAE values of between 3.5 days (Victoria) and 6.4 days (Conference) (Fig. 3).

**Table 3:** Best fitting parameters for each cultivar. ± values indicate standard deviation following the bootstrapping procedure

Parameter	Apple ‘Bramley’	Cherry ‘Stella’	Pear ‘Conference’	Plum ‘Victoria’
yc	70.34 ± 11.68	28.84 ± 2.48	32.40 ± 1.67	60.74 ± 3.15
zc	223.59 ± 46.13	165.93 ± 21.19	271.76 ± 9.13	122.08 ± 9.67
s1	0.99 ± 0.26	0.80 ± 0.19	1.00 ± 0.20	0.80 ± 0.14
Tu	17.82 ± 3.58	17.18 ± 1.19	16.62 ± 1.41	14.80 ± 0.35
E0	3209.96 ± 43.21	3348.03 ± 26.08	3391.97 ± 1.41	3369.83 ± 0.46
E1	9720.66 ± 47.32	9831.58 ± 25.91	9902.75 ± 1.06	9893.16 ± 0.58
A0	6021.25 ± 53.99	6067.81 ± 67.13	6092.49 ± 32.98	6673.28 ± 28.71
A1	5.93E+13 ± 1.90E+8	5.94E+13 ± 1.93E+8	5.94E+13 ± 2.49E+8	5.94E+13 ± 2.15E+8
Tf	6.55 ± 0.40	7.86 ± 1.63	7.13 ± 0.17	7.03 ± 0.76
Tc	20.59 ± 8.49	20.02 ± 2.62	42.94 ± 20.55	36.09 ± 7.62
Tb	6.87 ± 0.84	7.43 ± 0.87	3.90 ± 0.96	6.66 ± 0.23
slope	14.80 ± 4.07	10.32 ± 6.22	5.02 ± 2.03	4.32 ± 7.28

**Figure 4:**
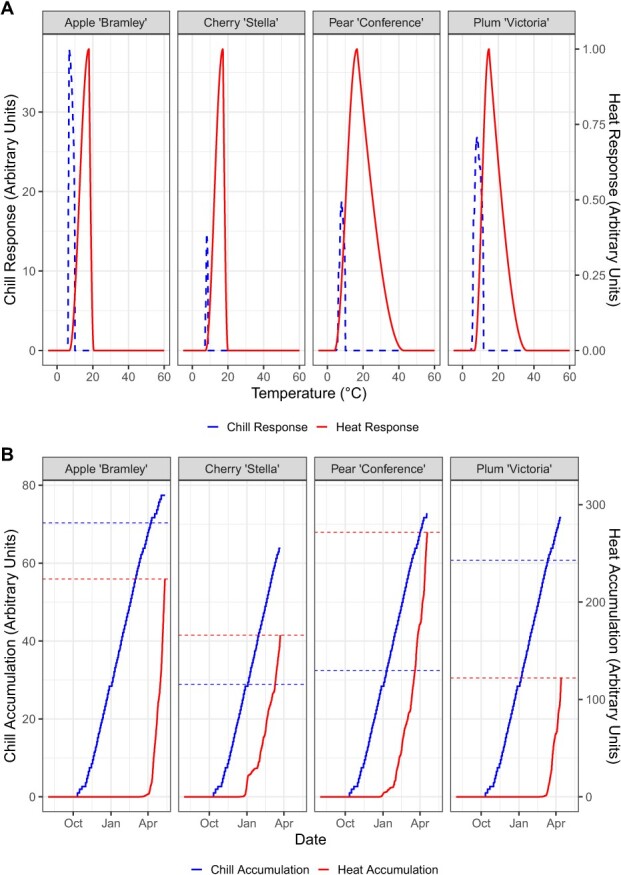
(A) Chill and heat response plots for each cultivar. The chill response (dashed line) shows chill effectiveness over a time period of 1200 hours assuming constant temperature. The heat response (solid line) represents heat efficiency for constant temperatures. (B) Example chill and heat accumulation curves for each cultivar, for the year 2022 in the grid square containing the National Fruit Collection. Solid lines indicate chill or heat accumulation, and dashed lines indicate thresholds yc (critical value of y, which defines the end of chill accumulation) and zc (critical value of z, which defines the end of heat accumulation).

Parameter values, and their uncertainty, used in the PhenoFlex modelling framework are presented in Table 3, and the values and uncertainty varied between cultivars. These parameters produced temperature response curves which appeared plausible for all four fruits, with chill response and accumulation stopping between 8°C and 12°C depending on the fruits, with optimum chilling temperatures around 7.5°C for all four fruits. Heat response and accumulation curves also appeared plausible, with optimum temperatures for all four fruits around 15°C–18°C (Fig. 4).

### How well can models parameterized using a single year of citizen science data predict flowering onset dates across space and time?

**Figure 5:**
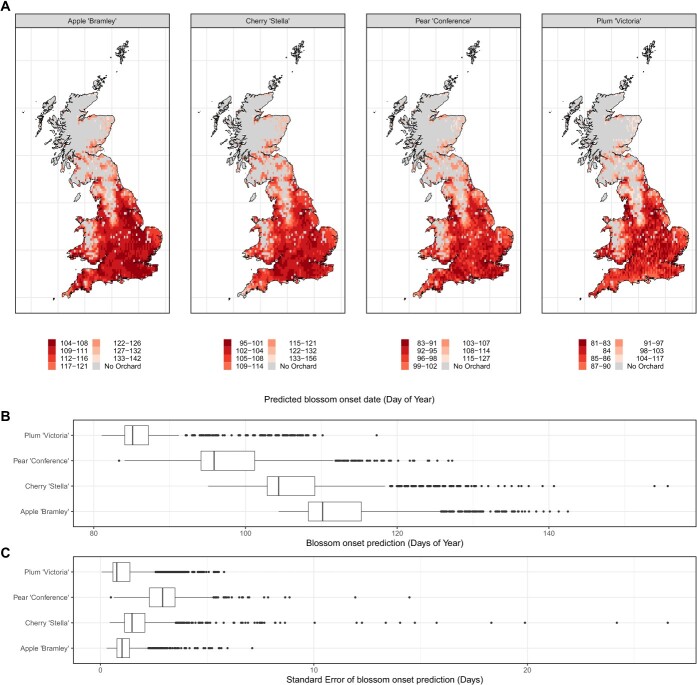
(A) Maps showing predictions of flowering onset dates for known orchard locations within Great Britain. Breaks were selected according to the Jenks natural breaks classification method [36]. (B) Boxplot showing distribution of flowering onset dates (the box highlights the main flowering onset period). (C) Boxplot showing distribution of standard errors of the flowering onset predictions, taken as the 16th and 84th percentiles of standard deviation of the 10 bootstrapping replications within each grid square.

Projections across known fruit-growing areas of Great Britain for all four fruits revealed earlier flowering onset in more southerly latitudes, and in urban areas, with the earliest flowering onset date for ‘Bramley’ predicted to occur in South-West London on April 14th (51°24′00.0″N 0°27′00.0″W). This was the same for ‘Stella’ (5th April—51°24′00.0″N 0°27′00.0″W), ‘Conference’ (24th March—51°24′00.0″N 0°33′00.0″W) and ‘Victoria’ (22nd March—51°36′00.0″N 0°33′00.0″W) (Fig. 5A). The flowering onset period varied between varieties, with ‘Victoria’ being the earliest, starting on March 25th and ending on March 28th. The flowering onset period was longer and later in the year for the other three varieties (6 days for ‘Stella’ and 7 days for ‘Bramley’ and ‘Conference’) (Fig. 5B). The uncertainty of these estimates also varied between varieties, with the lowest uncertainty produced in ‘Victoria’ with a median standard error of 0.77 days, compared with 1.02 days for ‘Bramley’, 1.49 days for ‘Stella’, and 2.91 days for ‘Conference’ (Fig. 5C).

Finally, models performed well when predictions were compared to records collected in a standardized manner from the National Fruit Collection. Between 1996 and 2022, the PhenoFlex model predicted ‘Bramley’ flowering onset occurring on average 3.6 days before recorded flowering onset at the NFC. For ‘Victoria’ and ‘Stella’ predictions were 4.8 days and 10.7 days later than recorded flowering onset dates, respectively. PhenoFlex predictions were 4.7 days earlier than recorded flowering onset dates for ‘Conference’ between 1996 and 2020.

## Discussion

It is well documented that citizen science recordings of the natural world may provide a good proxy for estimating dates of key phenological stages, and this study looks to utilize citizen science data to parameterize tree phenology models and ultimately to provide a national scale prediction of patterns of flowering onset across a flowering season.

### Does flowering onset phenology vary across a latitudinal gradient in Great Britain, across a single year?

Perhaps unsurprisingly, given the evidence from a study in the UK pointing towards later flowering onset phenology at higher latitudes [2], analysis of the FruitWatch submissions highlighted significant delays in flowering at more northerly sites for three of the four cultivars. It is well documented that temperature plays a large role in determining flowering dates [7] and given the gradient of temperature across Great Britian, with warmest temperatures generally in the South-West, gradually decreasing at increasingly northerly latitudes [23], this result is much to be expected.

### Can citizen science data be used to parameterize existing phenology models in a space-for-time substitution?

Here, RMSE values varied, ranging from 2.4 to 8.0 days. In comparison to similar studies utilizing the PhenoFlex framework, the citizen science informed models perform similarly to models collected through either standardized data collection schemes (RMSE 14–18 days in various Italian olive cultivars [24], experimental systems simulating different temperature conditions (RMSE 2.3–5.5 days in experimentally managed apple trees [25] or other proxies for estimating flowering (RMSE 4.56 days using airborne pollen data from the *Platanus* genus [26]. The models produced here also performed well when compared data collected from a standardized recording scheme at the National Fruit Collection, with a mean difference of 3.6–10.7 days between predicted and observed flowering dates over a 27-year period. Interestingly, the largest difference between predicted and observed dates was seen in ‘Stella’, which also had the fewest records, indicating that models perform better when more data is available.

The PhenoFlex framework is not the only modelling framework in existence for predicting flowering onset dates, and there are a range of different models that are commonly used. When considered alongside other fruit tree flowering onset models produced using different modelling frameworks, the RMSE values presented here were also comparable. Legave *et al*. [27] found RMSE values of between 3.6 and 5.3 days in western European ‘Golden Delicious’ apples using the Sequential Model, whereas Darbyshire *et al*. [28] found better performance using the Chill Overlap model in ‘Cripps Pink’ apples in Australia. These comparisons, therefore, provide a promising outlook for using citizen science data to parameterize relatively complex phenology models.

Visual inspections of the temperature response curves revealed an optimum chilling temperature of around 7.5°C for all four fruits. While this value is above the 7.2°C often used in the Chilling Hours concept [28], recent advances in the understanding of fruit tree phenology have shown that sharp thresholds are not always biologically relevant [29]. The 7.5°C optimum value reported here is around the values found in experimental systems on apple and cherry [30]. While the varieties differed between this study and the experimental study, the optimum chilling temperatures found here are in the same ballpark, again indicating that data generated through FruitWatch appears to be suitable for use with the PhenoFlex modelling framework. When considering the heat accumulation response, the optimum values for all four fruits are around 15°C, in keeping with the early spring temperatures around the flowering period, and again is similar to reported values from other studies [31].

### How well can models parameterized using a single year of citizen science data predict flowering dates across fruit-growing areas in that year?

A major benefit of citizen science data is that it can come from multiple locations, providing good geographic coverage. There is plenty of evidence showing variable flowering phenology in different locations, as well as variable phenology of pollinators and pests. With the models produced by the PhenoFlex framework shown to perform adequately, coupled with the geographic spread of records submitted to the FruitWatch platform, this study also looked to present a picture of the spatial variation in flowering onset dates across Great Britain for the 2022 flowering season.

By providing the models with temperature data for grid squares known to contain orchards, phenological gradients appear in multiple directions for all four fruits tested. Perhaps unsurprisingly given the well-established links between temperate fruit tree phenology and temperature, we see gradients related to latitude (delayed phenology at more northerly latitudes), elevation (delayed phenology at higher altitudes) and urbanization (advanced phenology in large urban areas such as London and Manchester).

The presence of urban heat islands (UHIs) is well established in Great Britain [32] and Great Britain is also seeing the UHI effect is increasing in intensity [33]. The effect of UHIs on phenology is also relatively well understood [34]. Jochner and Menzel [35] provide a comprehensive review of 45 studies on the phenology of a range of taxa in UHIs, and the majority of these studies show strong advances in phenological events in urban areas. In the context of fruit tree phenology, Roetzer *et al*. [36] show earlier flowering dates in urban compared with rural areas in central Europe in both apple and sweet cherry. Again, this evidence backs up the findings presented here, with clusters of early flowering onset concentrated around areas projected to suffer from the UHI effect [37] and can provide confidence that the predicted results fit with well-established phenological principles.

### Implications for growers

The suitability of FruitWatch (and more generally, citizen science) recordings for predicting flowering dates of fruit trees comes with a number of real-world applications, ranging from climate change detection to informing precision management of fruit crops and their pests and pollinators. It is well known that climate influences phenology of fruit trees [7] and fruit tree pollinators [38]. By building a long-term, spatially diverse set of records at the cultivar level, it is possible to detect shifts in local climates across a large scale.

While this study focusses explicitly on four types of fruit tree within Great Britain, it provides a framework for collecting citizen science data suitable for modelling spatial variation in flowering phenology. The tree selection can be expanded, to include fruits such as apricots and peaches, and the spatial scale of the FruitWatch platform can easily be increased, to accept records from a wider geographic range. With reports of declining winter chill coming from growing regions warmer than Great Britian, such as northern Africa [39] and California [40], coupled with future predictions of further declines in winter chill in the same studies, finding cultivars with suitable chill requirements for a given area is crucial for maintaining commercially viable orchards. The FruitWatch project aims to build an open access, long-term database of flowering dates of various fruit types and cultivars across a large spatial scale, and the flowering data generated through this project could help inform localized cultivar selection.

Given the importance of phenological synchrony between fruit tree flowering and pollination [17], the spatial understanding of flowering dates could be a vital tool in localized decision-making, which could influence, e.g. whether particular species of noncrop flowering plants are sown between rows in an orchard. This can be done to plug ‘hungry gaps’, or periods of low resource availability, by planting crops that flower at different periods to the main flowering period. Knowing when an orchard is likely to flower is also important if managed pollinators are to be introduced. Managed pollinators cost money to introduce to an orchard, costing US growers an estimated $350 million across all crops in 2009 [41], so understanding when they are likely to be needed could result in a better targeted—and more cost-effective—intervention.

While there can be confidence in the results presented here, both in terms of model parameterization and performance and spatial predictions, it is important to note that citizen science undeniably comes with challenges, especially opportunistic programs. Among the concerns are that participants often have no scientific background, that submissions are biased towards easily accessible locations and non-working days (i.e. weekends and public holidays) [42], and that taxa may be misidentified. However, in the case of this study, there did not appear to be large differences between the number of records submitted on each day, with fewer records submitted on Saturday than on every other day except Wednesday.

## Conclusion

The results presented here provide a promising outlook for the use of citizen science to inform flowering phenology models, meeting the three main questions set out at the start of this research. Firstly, with appropriate quality control checks, both in terms of the data being used and the models themselves, this study shows that citizen science data can be suitable for detecting latitudinal variation in flowering dates, as this study has shown significant shifts in flowering dates across a latitudinal gradient in three of the four cultivars studied. Secondly, this work also shows that phenology records submitted by citizen scientists can be a powerful data source for use in phenology modelling frameworks, with RMSE values comparable to those generated through standardized phenology recording programmes.

Looking to the future, the realistic predictions generated using the FruitWatch data give confidence that citizen science data can be a reliable source for making phenology predictions. In turn, this may inform growers of past, present, and, with enough temporal resolution (which was unfortunately not available for this study), potential future trends in phenology. With additional years data, FruitWatch (and similar recording schemes) could be used to support fine resolution and local decision-making across a national scale with relation to phenological synchrony with wild pollinators, and the timing of deployment of managed pollinators and pest and disease treatments.

## Materials and Methods

### The FruitWatch platform

The FruitWatch platform was built using Oracle Application Express (APEX) [43] and consists of a website (www.fruitwatch.org) containing project information and a simple recording form. The recording form consists of three stages. Firstly, information regarding the date and location of the record is collected. This can either be done automatically through a phone GPS, entering a postcode which is then converted to latitude and longitude, or through dropping a pin on a map. Secondly, information about the tree is collected. This involves recorders selecting the tree type (apple, pear, cherry, or plum) from a drop-down menu, and information about the cultivar (if known) is entered. At this point, recorders also input the phenological stage of the fruit tree, based on the well-established BBCH Scale for Pome and Stone Fruits [44]. This contains five categories: A (first flowers open, BBCH code 60), B (10% of flowers open, BBCH code 61), C (50% of flowers open, BBCH code 65), D (Flowers fading, BBCH code 67), and E (End of flowering, BBCH code 69). Finally, the recorders are asked to upload two photographs, firstly of the tree, and secondly of a cluster of flowers representative of the tree. This is currently an optional step and can be skipped if the recorder wishes to do so.

### Data cleaning

The recording scheme has been open since February 2022, and in that time 6696 records have been submitted. These records were passed through a filtering process. Initially, as uploading photographs was an optional step in the recording process, some records were uploaded without photographs, and as such the phenological stage could not be independently verified. Therefore, records without photographs were removed. Secondly, records from 19th March 2022 were removed. FruitWatch was featured in a national newspaper on this date, and as a result, 196 records were received on this day, potentially skewing the results towards this date. Next, records containing pictures without trees were removed, and records containing locations that were either outside Great Britain or with postcodes that could not be converted to latitude and longitude were removed.

Deciding which phenological stage a tree is at requires the recorder to make a subjective decision, and this can vary between recorders [45]. This is a common issue where subjective questions are included in citizen science, and recorder identity has been shown to explain almost 20% of the variance in vegetation percentage cover surveys, also a subjective measure [46]. Where records contain pictures, it is often possible to overcome this obstacle by having submitted records reclassified by a single person and comparing the consensus between the original records and the independent assessor.

To assess the accuracy of recordings, a random subsample of 1000 sets of photographs was assessed and reclassified by a single individual, a commonly used method for quality control in citizen science projects [47]. Of these 1000 records, 67.6% were classified as belonging to the same phenological stage by both the initial observer and the independent observer. 28% of results differed by only one phenological stage (i.e. A to B), with only 4.1% differing by more than one phenological stage (i.e. A to C), and the remaining 0.3% contained unusable images.

As a result of this lack of consensus between recorders and the independent verifier, especially when differentiating between adjacent phenological stages, records were reclassified into ‘Start’ (original codes A and B), ‘Full’ (original code C) and ‘End’ (original codes D and E). This resulted in a greater consensus of results, with 81.3% of results classified as the same by the original recorders and the independent recorder. This classification scheme was taken forward into further analyses, and the original recorders’ observations were used, excluding those with no or unusable pictures.

Finally, an outlier removal process was used. The interquartile range (IQR) method was used to identify outlying observations [48] within each cultivar and flowering stage. The IQR is calculated as the range between the 25th (Q_1_) and 75th (Q_3_) percentile values. Values lower than Q_1−_1.5^*^IQR or higher than Q_3_ + 1.5^*^IQR were removed.

Cultivar spelling and synonyms were standardized using a range of online sources, primarily from the databases of the National Fruit Collection (www.nationalfruitcollection.org.uk) and Pomiferous (www.pomiferous.com).

### Statistical analysis

#### Cultivar selection

As mentioned previously, apples, cherries, pears, and plums are amongst the most commonly grown fruit crops in Great Britian [5], and as a result, were chosen as focal crops for FruitWatch. Data for the most recorded cultivar of each fruit type for the year 2022 were selected for further analysis. This included the apple ‘Bramley’, cherry ‘Stella’, pear ‘Conference’ and plum ‘Victoria’. To model the spatial variation on flowering onset, records classed as ‘Onset’ (BBCH code 60 and 61—less than 10% flowers open, [44]) were selected for each cultivar. Where multiple records for a cultivar exist within the same grid square, the mean flowering date was calculated and used.

### Does flowering onset phenology vary across a latitudinal gradient in Great Britain, across a single year?

Using data for 2022 for the four selected cultivars, separate cultivar-level linear models were run, with latitude as the independent variable and recording date as the dependent variable to assess change in flowering onset date in relation to latitude. A Benjamini–Hochberg correction for multiple tests was applied to these models to help avoid Type I errors (q = 0.05) [49].

### Can citizen science data be used to parameterize existing phenology models in a space-for-time substitution?

Fruit trees’ relationship with temperature is somewhat complex, and trees require exposure to cool temperatures (known as the ‘chilling’ period), followed by warm temperatures (known as the ‘forcing’ period) to break dormancy. A recently developed modelling framework, called ‘PhenoFlex’ [31] incorporates two commonly used models to predict bloom dates. It uses the dynamic model [50] to account for chill accumulation and the growing degree hours model [51] to account for heat accumulation. A detailed description of the PhenoFlex framework can be found in [31], and this framework has performed well in predicting flowering dates in temporal phenology series [31].

The PhenoFlex framework requires hourly maximum and minimum temperature data, and so daily minimum and maximum temperature for this period between 1 January 2021 and 30 June 2023 were obtained at 0.1° gridded resolution from the ensemble mean of the E-Obs database version 27.0e [52] and downscaled using the *stack_hourly_temps* function within the chillR package [53]. Each phenology submission was then assigned to its corresponding grid square, so it could be linked to temperature records.

The PhenoFlex framework allows for many parameters (Table 4) to be set which allows for high flexibility when forecasting phenology across different cultivars. Each cultivar was analysed separately, allowing for cultivar-specific parameter estimates.

**Table 4:** List and descriptions of parameters used in the PhenoFlex modelling framework, and their initial values used in the parameter fitting procedure

Parameter	Description	Starting value *(lower, upper)*
yc	Chilling requirement: critical value of y, which defines the end of chill accumulation	40 *(20, 80)*
zc	Heating requirement: critical value of z, which defines the end of heat accumulation	190 *(100, 500)*
s1	Slope parameter that determines the transition from the chill accumulation to the heat accumulation period in PhenoFlex	0.5 *(0.1, 1.0)*
Tu	Optimal temperature of the growing degree hours (GDH) model	25 (*0, 30)*
E0	Time-independent activation energy of forming the precursor to the dormancy-breaking factor (PDBF)	3372.8 *(3000.0, 4000.0*)
E1	Time-independent activation energy of destroying the precursor to the dormancy-breaking factor (PDBF)	9900.3 *(9000.0, 10000.0)*
A0	Amplitude of the (hypothetical) process involved in forming the precursor to the dormancy-breaking factor in the dynamic model	6319.5 (*6000.0, 7000.0)*
A1	Amplitude of the (hypothetical) process involved in destroying the precursor to the dormancy-breaking factor (PDBF) in the dynamic model	5.939917e13 *(5e13, 6e13)*
Tf	Transition temperature parameter of the sigmoidal function in the dynamic model, also involved in converting the PDBF to chill portions	4 *(0, 10)*
Tc	Upper threshold in the GDH model	36 *(0, 40)*
Tb	Base temperature of the GDH model	4 *(0, 10)*
slope	Slope parameter of the sigmoidal function in the dynamic model, which determines what fraction of the PDBF is converted to chill portions.	1.60 *(0.05, 50.00)*

Initially, the dataset was ordered by latitude and split into calibration and validation subsets. 75% of records for each cultivar were used to calibrate the model, with the remaining 25% used for validation, in a repeating pattern of ‘v’, ‘c’, ‘c’, ‘c’ (where ‘v’ = validation, ‘c’ = calibration). This was split to capture a range of latitudes, and therefore temperature profiles, within both the calibration and validation datasets.

The GenSA algorithm [54] was used to parameterize the PhenoFlex model, using the calibration dataset as phenological records, using the starting values and parameter ranges set in Table 4, these parameters are deliberately wide following initial parameters bounds used in similar PhenoFlex studies [25]. A maximum of 1000 iterations of the algorithm were run, and the process was stopped when there was no further improvement in model fit after 250 consecutive iterations.

To assess the suitability of the citizen science phenology recordings for use in the PhenoFlex framework, and for making predictions of flowering dates, the model for each cultivar was evaluated by calculating the root mean square error (RMSE) and mean absolute error (MAE) of both the calibration and validation datasets. As an additional step, temperature response curves (chill and heat accumulation) were fitted using the final parameters and visually inspected for plausibility.

In similar studies, the best-fitting parameters were only obtained after multiple iterations of the optimization procedure [25]. Therefore, the optimization process was run multiple times in an attempt to further refine models and reduce error. The parameter values were changed in each iteration to reflect the values provided by the previous iteration. This process of refining only stopped after two consecutive unsuccessful iterations (i.e., no improvement in RMSE or MAE), and the parameter estimates from the final model showing improvement were taken forwards as the best parameter estimates.

Standard errors of the best parameter estimates were calculated using a bootstrapping technique, which was repeated 10 times. Bootstrapping was carried out following the methods described by Fernandez *et al*. [25] and Luedeling *et al*. [31], as Fernandez *et al*. [25] describe it, it involves randomly sampling the residuals for the flowering onset dates calculated during the calibration phase of the PhenoFlex model and adding the randomly sampled residuals to the original flowering onset dates, effectively creating a new dataset. Secondly, the parameter fitting procedure was re-run, generating a new set of parameters. This procedure was repeated ten times, and the standard deviation across the bootstrapping iterations was calculated as a measure of uncertainty in the parameter estimates. The 16th and 84th percentiles of the standard deviation were also calculated, which provide estimates of the standard error. The flowering onset dates for the validation dataset were estimated using the parameters generated by each of the ten bootstrapped replicates, and uncertainty was expressed as the standard deviation of the ten replications.

### How well can models parameterized using a single year of citizen science data predict flowering onset dates across space and time?

To attempt to understand how flowering onset phenology changes across Great Britain in a single year, the parameter estimates generated in the previous section were used to estimate flowering onset dates for grid squares without records. Temperature data for grid squares known to contain orchards, based on a map of known orchard locations in 2016, from the Ordnance Survey MasterMap (www.ordnancesurvey.co.uk), were extracted, and converted to hourly series using the *stack_hourly_temps* function from the chillR package [53] as described previously.

Within each grid square known to contain an orchard, flowering dates for each of the four cultivars were predicted by running the PhenoFlex modelling framework with weather data for each grid square and the best parameter estimates generated through the previously described fitting procedure. To express uncertainty in the flowering onset estimation, predictions were also made using the parameters generated from each of the ten bootstrapping iterations. The standard deviation across the predictions for the ten bootstrapping iterations was calculated as well as the 16th and 84th percentiles of the standard deviation to provide estimates of the standard error. The ‘flowering onset period’ was also calculated for each cultivar, as being between the 25th and 75th percentile predicted flowering onset dates.

As an additional measure of model performance and to assess the models’ ability to predict flowering onset dates over time, predictions were compared to flowering onset records collected in a standardized manner from the National Fruit Collection (NFC), held in Faversham, Kent. To do this, daily maximum and minimum temperatures for the grid square where the NFC is located were extracted for the period 1995–2022. Following the same steps as above, flowering onset phenology was predicted for each fruit in the grid square containing the NFC for the period 1996–2022. Actual flowering onset records from the NFC were obtained for the period 1996–2022 for ‘Bramley’, ‘Stella’, and ‘Victoria’ and 1996–2020 for ‘Conference’ and the difference (in days) between the PhenoFlex predictions and the NFC records was calculated.

## Acknowledgements

The authors would like to thank Ajay Kumar, for his advice and support developing www.fruitwatch.org, the many University of Reading students and staff who contributed to the testing of FruitWatch and all the members of the public who have submitted records. This project was funded by BBSRC (Grant number: BB/T508895/1) and Waitrose Agronomy Group as part of the Waitrose Collaborative Training Partnership. This work was supported in part by Oracle Cloud credits and related resources provided by the Oracle for Research program (Grant number: 16366771).

## Data availability

The data that support the findings of this study are openly available in The University of Reading Data Archive. Available at: https://doi.org/10.17864/1947.000524

## Conflict of interest statement

The authors declare no conflict of interest.
